# Natural Course of Asymptomatic Upper Gastrointestinal Subepithelial Lesion of 2 cm or Less in Size

**DOI:** 10.3390/jcm11247506

**Published:** 2022-12-18

**Authors:** Donghyun Kim, Seoyeon Cho, Seon-Young Park, Hye-Su You, Yong-Wook Jung, Su-Hyeon Cho, Changhwan Park, Hyun-Soo Kim, Sungkyu Choi, Jongsun Rew

**Affiliations:** Division of Gastroenterology, Department of Internal Medicine, Chonnam National University Hospital and Medical School, Gwangju 61706, Republic of Korea

**Keywords:** subepithelial lesion, endoscopy, endoscopic ultrasonography, prognosis

## Abstract

There is limited evidence of a natural course of an upper gastrointestinal (UGI)-subepithelial lesion (SEL) of 2 cm or less in size. This study aims to determine the natural course of UGI-SELs and find the risk factors of the endoscopic and endoscopic ultrasonography (EUS) findings associated with an increase in size. The medical records of 2539 patients with UGI-SELs between 2004 and 2016 were reviewed retrospectively. A total of 672 SELs of 2 cm or less in size were analyzed through EUS and followed up for at least 36 months. The mean follow-up duration was 68 months (range: 36–190 months), and 97 SELs (14.4%) showed an increase in size with a mean increase rate of 1.2 mm/year. Initial size (aOR 1.03, 95% confidence interval (CI) 1.01–1.06), an endoscopic finding of a hemorrhagic spot (aOR 3.13, 95% CI 1.14–8.60), and an EUS finding of a lesion in the fourth layer (aOR 1.87, 95% CI (1.21–2.88) were related to an increase in size. An endoscopic finding of translucidity (aOR 0.28, 95% CI (0.10–0.76) and an EUS finding of calcification (aOR 0.30, 95% CI 0.09–0.95) were inversely related to an increase in size. There was no death related to UGI-SELs during the follow-up. While most UGI-SELs of 2 cm or less in size showed no significant size change and favorable prognosis, an individualized follow-up strategy needs to be considered in case of the presence of hemorrhagic spots and lesions in the fourth layer.

## 1. Introduction

Subepithelial lesions (SELs) of the upper gastrointestinal (UGI) tract include a variable differential diagnosis, which covers benign and malignant tumors. Endoscopic ultrasonography (EUS) can be beneficial to the differential diagnosis of SELs and decisions on management strategies because it provides useful information regarding the location, size, and echogenic characteristics of tumors [1,2]. Recently, the use of artificial intelligence has been suggested as a less-invasive approach for endoscopic diagnosis including UGI-SELs with better diagnostic accuracy to differentiate potential malignant lesions from other subepithelial lesions [3,4,5,6,7].

In some instances, endoscopists need to decide whether to perform a surgical or endoscopic procedure for removal or EUS-guided intervention to sample tissues, especially in atypical cases or lesions with a malignant potential [8,9,10,11,12,13]. However, resection is not recommended for most UGI-SELs considered to be benign tumors without any clinical symptoms. A serial follow-up can be more appropriate if the size of UGI-SELs seems stable [14,15,16]. According to the European Society of Gastrointestinal Endoscopy guideline, EUS-guided sampling is not recommended for <2 cm SELs located in the esophagus or stomach, and a follow-up is recommended [17]. However, with respect to a follow-up, clear consensus on interval and period is lacking [18,19,20].

Thus far, long-term follow-up data for benign-looking UGI-SELs and studies on the possible effect of an increase in size of SELs on a patient’s prognosis are lacking [21,22]. In the present study, we aimed to evaluate the natural course of a benign-looking UGI-SEL 2 cm or less in size and the risk factors of a baseline endoscopic or EUS finding for predicting the increase in size.

## 2. Materials and Methods

### 2.1. Patients

**Figure 1:**
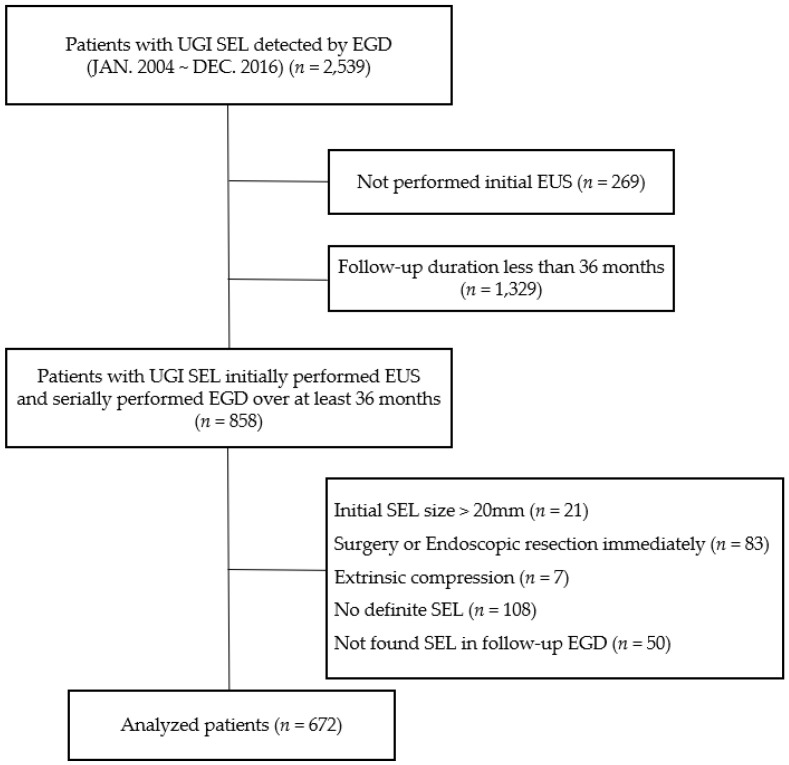
Flow chart of the enrolled patients. UGI, upper gastrointestinal; SEL, subepithelial lesion; EGD, esophagogastroduodenoscopy; EUS, endoscopic ultrasonography.

This study was a retrospective observational study. From January 2004 to December 2016, UGI-SELs were identified in 2539 patients through esophagogastroduodenoscopy (EGD) at Chonnam National University Hospital, Gwangju, Korea. The following patients were included: (1) patients who underwent EUS for the evaluation of UGI-SEL and (2) patients who had serial EGD to check the change in the size of SEL for at least 36 months. The following patients were excluded: (1) patients with >2 cm SELs, (2) patients who underwent surgical or endoscopic resection immediately after initial EUS because of malignant potential, (3) patients who suggested extrinsic compression or no definite SEL in EUS finding, and (4) patients without definite SEL in the follow-up EGD. A total of 672 patients constituted the study population who met the inclusion and exclusion criteria (Figure 1). Of these patients, 103 had multiple UGI-SELs, and only the largest SEL was analyzed as a representative lesion. Therefore, 672 UGI-SELs were analyzed in 672 patients.

This study was conducted in accordance with the ethical guidelines of the Declaration of Helsinki. The study protocol was approved by the ethics committee of the Chonnam National University Hospital (Institutional Review Board Number: CNUH-2020-116; approval date: 5 July 2020).

### 2.2. Endoscopic and EUS Evaluation

**Figure 2:**
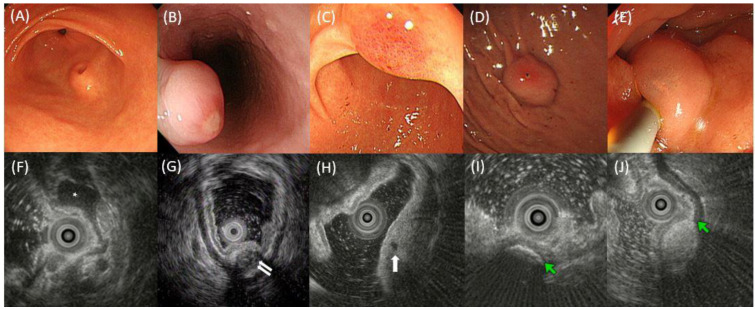
Examples of the endoscopic and EUS findings of SELs. (**A**) Umbilication or central dimpling, (**B**) erosion or ulcer, (**C**) erythema, (**D**) hemorrhagic spot, and (**E**) translucidity. (**F**) EUS image showing a homogeneous hypoechoic SEL (asterisk) with a distinct border; (**G**) EUS image showing an SEL involving muscle propria layer (white arrows) with heterogeneity, mixed echogenicity, indistinct border, and deep attenuation; (**H**) EUS image showing anechoic foci (white arrow); (**I**) EUS image showing hyperechoic foci (green arrow) with acoustic shadowing that suggests calcification; (**J**) EUS image showing an SEL involving submucosal layer (green arrow indicates hyperechoic submucosal layer) with hyperechoic echogenicity, homogeneity, and distinct border. EUS, endoscopic ultrasonography; SEL, subepithelial lesion.

All examinations were performed by experienced endoscopists (each with experience of >3000 endoscopic examinations). SEL was measured using a visual estimate of the largest diameter of open biopsy forceps during endoscopy as a size criterion (open diameter of 8.0 mm; reusable type, Olympus, Tokyo, Japan). Endoscopic and EUS findings were assessed independently by two endoscopists (DH Kim and SY Cho). Endoscopic and EUS findings were re-evaluated in case of disagreement between the two endoscopists, who reviewed the findings and then reached an agreement. The following endoscopic features were recorded: (a) initial size, (b) presence of umbilication or central dimpling on the surface, (c) presence of erosion or ulcer, (d) presence of erythema, (e) presence of hemorrhagic spot, and (f) presence of translucidity (Figure 2). EUS probes (UM-2R/UM-3R: Olympus) and a probe-driving unit (MH-MAJ-1720: Olympus) were initially used to map the lesions. The imaging frequency of the probe was 12 or 20 MHz. EUS was performed by experienced endosonographers (DH Kim, SY Park, and JS Rew). Lesions were scanned after the UGI tract was filled with distilled water. The following EUS features were also recorded: (a) maximal and cross-sectional diameters, (b) layer of origin, (c) homogeneity (homogeneous or heterogeneous), (d) distinctness of the border (distinct or indistinct), (e) presence of anechoic foci (duct-like structure), (f) presence of hyperechoic foci with acoustic shadowing (suggesting calcification), (g) deep attenuation, and (h) presence of septation (Figure 2). SELs were classified as superficial (S-type) and deep (D-type) types that were modified based on the proposed classification proposed by Park et al. [23]. In the S-type, lesions originated in the second and/or third layer. In the D-type, lesions were found in the fourth layer with or without extension into the fifth layer.

### 2.3. Endpoints

The primary endpoint was to analyze predictors for an increase in size of UGI-SELs. The secondary endpoint was to identify the mortality rate associated with an increase in size of SEL. An increase in size of UGI-SEL was defined as a 25% increase in the longest diameter of SEL upon endoscopic inspection.

### 2.4. Analysis of Survival

Patients’ death, date of death, and death-related disease codes were confirmed by requesting data from the Korea National Statistical Agency (http://kostat.go.kr/portal/eng/index.action, accessed on 12 October 2020). Disease codes were classified in accordance with the Korean Standard Classification of Disease and Cause of Death 7 of the Korea Informative Classification of Diseases [24,25].

### 2.5. Statistical Analysis

Data were statistically analyzed using SPSS version 25.0 (Armonk, NY, USA: IBM Corp). For continuous variables, differences between groups were evaluated using an unpaired *t*-test. For discrete variables, differences were expressed as counts and percentages and analyzed using a χ^2^ test or Fisher’s exact between groups as appropriate. The increase in size of UGI-SELs was subjected to Kaplan–Meier analysis, and differences among the groups were assessed via a log-rank test. Statistical differences between groups were analyzed via a pairwise over strata method. Cox proportional hazard regression was performed to analyze the adjusted odds ratios (aORs) as estimates of the increase in size of UGI-SELs. Relevant variables in regression analysis were controlled. All potentially relevant variables were as follows: age, sex, and baseline characteristics of endoscopic and EUS findings. Statistical significance was set at *p* < 0.05.

## 3. Results

A total of 672 patients with UGI-SELs (mean age of 54.6 ± 10.7 years; 278 males, 41.4%) were included in the final analysis. The mean initial diameter of the SELs was 10.7 ± 4.1 mm (range of 3–20 mm). The location of the SELs was the esophagus in 152/672 (22.6%) patients, stomach in 430/672 (64.0%) patients, and duodenum in 97/672 (14.4%) patients. During the median follow-up of 68 months (range of 36–190 months), 97 (14.4%) patients had an increase in size of UGI-SELs. The mean size increment was 6.78 ± 4.20 mm (median of 5 mm). The annual increase in size (mean ± SD) was 1.21 ± 0.93 mm/year (median of 0.95 mm/year). Of the 97 patients with an increase in size of UGI-SEL, 46 patients had UGI-SELs with over 20 mm in size (initial mean size of 14.9 ± 3.13 mm; median of 15 mm).

Table 1 shows the differences in the demographic, endoscopic, and EUS findings between the patients without an increase in size of UGI-SELs and the patients with an increase in size of UGI-SELs. There was a difference in age at the time of diagnosis of UGI-SEL between patients without an increase in size (54.2 ± 10.6 years) and patients with an increase in size (56.8 ± 10.6 years, *p* = 0.03). An increase in size of UGI-SELs was more frequently observed in the stomach than in the esophagus or duodenum (*p* < 0.01). Endoscopic findings such as erythema (*p* < 0.01) and hemorrhagic spots (*p* = 0.04) significantly differed between the two groups. Translucidity tended to be less frequently observed in patients with an increase in size (*p* = 0.06). EUS findings such as longitudinal diameter (*p* < 0.01), cross-sectional diameter (*p* < 0.01), and D-type (*p* = 0.01) also significantly differed between the two groups.

### 3.1. Cox Regression Analysis to Predict an Increase in Size of UGI-SELs

Among endoscopic findings, initial size (aOR = 1.03, 95% confidence interval (CI) 1.01–1.06, *p* = 0.03) and hemorrhagic spots (aOR = 3.13, 95% CI 1.14–8.60, *p* = 0.03) were related to an increase in size of UGI-SELs (Table 2). Presence of translucidity was inversely related to an increase in size of UGI-SELs (aOR = 0.28, 95% CI = 0.10–0.76, *p* = 0.01).

Among EUS findings, longitudinal diameter (aOR 1.11, 95% CI 1.06–1.16, *p* < 0.01) and deep type (aOR 1.87, 95% CI 1.21–2.88, *p* < 0.01) were related to an increase in size of UGI-SELs (Table 3). The presence of calcification was inversely related to an increase in size of UGI-SELs (aOR = 0.30, 95% CI = 0.09–0.95, *p* = 0.04).

### 3.2. Clinical Course of Patients with an Increase in Size of UGI-SELs

Among the 97 patients with an increase in size of UGI-SELs, 32 (33.0%) patients underwent surgical resection, 14 (14.4%) patients underwent endoscopic resection, and 1 patient (1.0%) underwent EUS-guided fine-needle tissue acquisition for pathologic confirmation. Among the pathologic findings based on the histology and immunohistochemistry of 47 patients, gastrointestinal stromal tumor (GIST; 27/47, 57.4%) was the most common, followed by leiomyoma (5/47, 10.6%) and lipoma (4/47, 8.5%). The 50 remaining patients (51.5%) were followed up with annual EGD: UGI-SEL diameter of less than 20 mm (*n* = 42), an EUS finding suggesting a lipoma (*n* = 5), and refusal of further diagnostic work-up or surgical resection (*n* = 3).

### 3.3. Survival Analysis

The result of the Korea National Statistical Agency showed that 16 patients (16/672, 2.4%) died. The median follow-up duration was 120 months (range of 44–193 months). Of the patients with an increase in size of UGI-SELs, 4 patients (4/97, 4.1%) died. Of the patients without an increase in size of UGI-SELs, 14 patients (12/575, 2.1%) died. The most common cause of death was lung cancer (*n* = 3), followed by pneumonia (*n* = 2) and trauma (*n* = 2). The malignancy of the UGI tract was not a cause of death in all patients who died.

## 4. Discussion

In our study, patients’ age and the size of UGI-SELs at the time of diagnosis was related with an increase in size of UGI-SELs. Endoscopic finding of hemorrhagic spot and EUS findings of the deep type (lesion in the fourth layer) were related with an increase in size, whereas an endoscopic finding of translucidity and EUS findings of anechoic foci and calcification were inversely related with an increase in size of UGI-SELs. There was no UGI-SELs-related death in patients with UGI-SELs of 2 cm less in size.

The size of UGI-SELs increased in 14.4% of patients with small UGI-SELs (<2 cm in size), which were higher than that described in previous similar studies (3–9%) [26,27,28]. These differences were attributed to the longer follow-up period of our study (median 68 months) compared with those in other studies [26,27,28]. The frequency of the increase in the size of UGI-SELs was higher in the stomach (17.7%) than in the esophagus (7.9%) or duodenum (10.0%). This finding was similar to previous results [27,28]. In our study, age at the initial diagnosis was positively correlated with an increase in size of UGI-SELs, which was similar to Song’s study [27].

Gross endoscopic findings associated with an increase in size of UGI-SELs included the initial size and hemorrhagic spot. Hemorrhagic spots of SELs may be a stigma of recent bleeding [27,28]. Gross endoscopic findings such as of translucidity were inversely related with an increase in size of UGI-SELs. The translucidity of SELs may indicate benign cystic components of lymphangioma or cyst [13,25,26].

Limited information is available about the characteristic EUS findings associated with the natural course because of the relatively short follow-up duration and the small number of cases [21,22]. Kim et al. [26] reported that any EUS findings were not related to an increase in size of SELs in 476 patients during the follow-up of 30 months. In the present study, EUS findings such as D-type and absence of anechoic foci or calcification were related to an increase in size of UGI-SELs. In EUS, GIST is often a hypoechoic lesion invading the muscle propria (fourth) layer. Benign SELs (such as Brunner’s gland hyperplasia, lipoma, cyst, and ectopic pancreas) often originate from the second or third layers [29]. This difference likely explains why the D-type SEL has a higher rate of size increase than the S-type SEL. Calcification is more commonly observed in leiomyoma (6.5–18%) compared with GIST and schwannoma (0–3.5% and 0–3.7%, respectively) [29]. Since leiomyoma has a relatively low rate of increase in size compared with that of GIST or schwannoma, an increase in size may be less frequently observed in the presence of calcification in SELs. Anechoic foci are commonly observed in benign lesions such as ectopic pancreas [30].

Using the databases of the Korea National Statistical Agency, we found 16 (2.4%) patients who died. Among them, no one died because of UGI cancer, suggesting favorable prognosis.

Our study included the large number of patients with long-term follow-up durations among studies regarding the natural course of small UGI-SELs. While most UGI-SELs of 2 cm or less in size showed no significant size change and favorable prognosis, individualized follow-up strategies need to be considered in cases of the presence of hemorrhagic spots and lesions in the fourth layer.

This study had some limitations. First, it was a single-center single-nation study. Second, in this retrospective study, data were not collected prospectively. Therefore, observation errors might have occurred even though we tried to reduce them. Nonetheless, long-term follow-up data of numerous patients were obtained, and they had a high value as EUS was initially performed. Therefore, our study of UGI-SELs of 2 cm or less in size support the current practice of observing, rather than the histological assessment or removal of such lesions. More clear facts related to the initial EUS finding and the change in the size of SELs should be obtained through subsequent studies.

## 5. Conclusions

While most UGI-SELs less than 2 cm in size showed no significant size change and favorable prognosis, individualized follow-up strategies need to be considered in cases of the presence of hemorrhagic spots and lesions in the fourth layer.

## Figures and Tables

**Table 1:** Demographic, endoscopic and ultrasonographic findings of 672 patients with upper gastrointestinal subepithelial lesions.

	All Patients (*n* = 672)	Patients without an Increase in Size (*n* = 575)	Patients with an Increase in Size (*n* = 97)	DF	*p-*Value
Age, years	54.6 ± 10.7	54.2 ± 10.6	56.8 ± 10.6	670	0.03
Male	278 (41.4)	233 (40.5)	45 (46.4)	1	0.28
Initial size, mm	10.7 ± 4.1	10.5 ± 4.1	11.7 ± 4.2	670	0.01
Location				2	<0.01
Esophagus	152 (22.6)	140 (24.3)	12 (12.4)		
Stomach	430 (64.0)	354 (61.6)	76 (78.4)		
Duodenum	90 (13.4)	81 (14.1)	9 (9.3)		
Endoscopic finding					
Umblication	35 (5.2)	31 (5.4)	3 (3.1)	1	0.60
Erosion or ulcer	37 (5.5)	31 (5.4)	6 (6.2)	1	0.75
Erythema	52 (7.7)	38 (6.6)	14 (14.4)	1	<0.01
Hemorrhagic spot	11 (1.6)	7 (1.2)	4 (4.1)	1	0.04
Translucidity	63 (9.4)	59 (10.3)	4 (4.1)	1	0.06
EUS findings					
Longitudinal diameter, mm	9.4 ± 4.1	9.1 ± 4.1	10.7 ± 4.0	670	<0.01
Cross sectional diameter, mm	6.6 ± 2.9	6.4 ± 2.8	7.9 ± 3.2	670	<0.01
Deep type	371 (55.2)	306 (53.2)	65 (67.0)	1	0.01
Heterogeneity	102 (15.2)	92 (16.0)	10 (10.3)	1	0.15
Distinct border	616 (91.7)	524 (91.1)	92 (94.8)	1	0.22
Anechoic foci	51 (7.6)	46 (8.0)	5 (5.2)	1	0.33
Caclfication	50 (7.4)	47 (8.2)	3 (3.1)	1	0.08
Deep attenuation	33 (4.9)	31 (5.4)	2 (2.1)	1	0.16
Septation	34 (5.1)	30 (5.2)	4 (4.1)	1	0.65

Data are shown as mean ± standard deviation or *n* (%); EUS, endoscopic ultrasonography; DF, degree of fre.0.edom.

**Table 2:** Cox regression analysis of endoscopic findings to predict an increase in the size of upper gastrointestinal subepithelial lesions.

Variable	DF	cOR (95% CI)	*p*-Value	DF	aOR (95% CI)	*p-*Value
Age, year	1	1.03 (1.01–1.05)	0.01	1	1.03 (1.01–1.06)	<0.01
Male	1	1.06 (0.70–1.61)	0.79			
Initial size, mm	1	1.06 (1.01–1.11)	0.03	1	1.06 (1.01–1.11)	0.03
Umblication	1	0.54 (0.20–1.20)	0.24			
Erosion or ulcer	1	1.79 (0.66–4.90)	0.25			
Erythema	1	1.66 (0.84–3.30)	0.15			
Hemorrhagic spot	1	3.36 (1.10–10.29)	0.03	1	3.13 (1.14–8.60)	0.03
Translucidity	1	0.27 (0.10–0.75)	0.01	1	0.28 (0.10–0.76)	0.01

cOR, crude odds ratio; CI, confidence interval; aOR, adjusted odds ratio; DF, degree of freedom.

**Table 3:** Cox regression analysis of endoscopic ultrasonographic findings to predict an increase in the size of upper gastrointestinal subepithelial lesions.

Variable	DF	cOR (95% CI)	*p*-Value	DF	aOR (95% CI)	*p-*Value
Age, year	1	1.04 (1.02–1.06)	<0.01	1	1.04 (1.02–1.06)	<0.01
Male	1	1.06 (0.70–1.61)	0.79			
Longitudinal diameter, mm	1	1.13 (1.07–1.18)	<0.01	1	1.11 (1.06–1.16)	<0.01
Deep type	1	1.64 (1.04–2.60)	0.03	1	1.87 (1.21–2.88)	0.01
Heterogeneity	1	1.15 (0.48–2.76)	0.76			
Distinct border	1	0.79 (0.29–2.17)	0.65			
Anechoic foci	1	0.38 (0.12–1.19)	0.10			
Caclfication	1	0.31 (0.10–1.01)	0.05	1	0.30 (0.09–0.95)	0.04
Deep attenuation	1	0.51 (0.12–2.19)	0.37			
Septation	1	0.44 (0.15–1.23)	0.14			

cOR, crude odds ratio; CI, confidence interval; aOR, adjusted odds ratio; DF, degree of freedom.

## Data Availability

The data are not publicly available because of privacy and ethical restrictions. The data presented in this study are available upon request from the corresponding author.
